# The Effects of Potato Presentation on Vegetable Intake in School-Aged Children: A Cross-Over Study

**DOI:** 10.3390/nu15214496

**Published:** 2023-10-24

**Authors:** Mayra G. Hernandez Sanchez, Sarah Bellini, William F. Christensen, Laura K. Jefferies, James D. LeCheminant, Emily V. Patten, Alisha H. Redelfs, Nathan Stokes, Jacklyn Wang, Micaela Rennick, Kelsey Anderson, Joli Hunt, Gene J. Ahlborn

**Affiliations:** 1 Department of Nutrition, Dietetics and Food Science, Brigham Young University, Provo, UT 84602, USA; mayrag007@gmail.com (M.G.H.S.); sarah_bellini@byu.edu (S.B.); laura_jefferies@byu.edu (L.K.J.); james_lecheminant@byu.edu (J.D.L.); emily_patten@byu.edu (E.V.P.); nathan_stokes@byu.edu (N.S.); jwangbyu@gmail.com (J.W.); micaelarennick@gmail.com (M.R.); kelseyrperry@gmail.com (K.A.); huntjoli@gmail.com (J.H.); 2 Department of Statistics, Brigham Young University, Provo, UT 84602, USA; william@stat.byu.edu; 3 Department of Public Health, Brigham Young University, Provo, UT 84602, USA; alisha_redelfs@byu.edu

**Keywords:** school nutrition, vegetable consumption, children’s nutrition, school meals

## Abstract

Vegetables are an essential component of a healthy dietary pattern in children; however, their consumption is often insufficient due to lack of preference. To address this, the influence of combining vegetables (mixed peas and carrots—MPACs) with potatoes, a generally liked food, on overall vegetable consumption among children aged 7–13 years was explored. The research involved a cross-over study design with 65 participants who completed five lunchtime meal conditions, each with different combinations of MPACs and potatoes versus a control (MPACs with a wheat roll). The meals were provided in a cafeteria setting, and plate waste was used to measure vegetable consumption. Anthropometric data and other variables were also measured. Notably, self-reported hunger did not significantly differ between conditions. Meal condition was a significant predictor of MPACs (F = 5.20; *p* = 0.0005), with MPAC consumption highest when combined with shaped potato faces in the same bowl (+8.77 g compared to serving MPACs and shaped potato faces in separate bowls) and lowest when combined with diced potatoes in the same bowl (−2.85 g compared to serving MPACs and diced potatoes in separate bowls). The overall model for MPAC consumption was influenced by age, height z-score, body fat percentage z-score, and condition (likelihood ratio = 49.1; *p* < 0.0001). Age had the strongest correlation with vegetable consumption (r = 0.38), followed by male gender, height z-score (r = 0.30), and body fat z-score (r = −0.15). The results highlight the positive impact of combining potatoes with vegetables in school meals, particularly when using shaped potato faces. These findings emphasize the potential of potatoes as a valuable vegetable option in promoting healthier eating habits among children. Additionally, future research could explore the impact of different potato combinations and investigate other factors influencing meal consumption in school settings.

## 1. Introduction

Dietary patterns are known to influence the risk of chronic diseases such as coronary artery disease, hypertension, and diabetes [1,2,3,4,5,6]. In addition, the consumption of a healthy diet is positively associated with both physical and mental health [7]. Since patterns of eating are often developed early in life, the promotion of healthy eating among children is essential [8,9].

Vegetables are a crucial component of a healthy dietary pattern [10,11]. They provide the essential nutrients that are needed for children to grow and develop properly. The 2020 to 2025 Dietary Guidelines for Americans recommend 1 to 2.5 vegetable cup equivalents for children aged 2 to 8 years, 1.5 to 3.5 vegetable cup equivalents for children aged 9 to 13 years, and 2.5 to 4 vegetable cup equivalents for adolescents aged 14 to 18 years [12]. While 91% of children aged 2–19 years report consuming at least one vegetable cup equivalent per day, few children achieve the recommended intake of vegetables [13,14].

Vegetable consumption in children may be positively influenced in multiple ways, such as through parental modeling, educational games, family interventions, and school interventions [15,16,17]. In addition, repeated exposures to vegetables and increasing portions of vegetables served are among tactics that may positively affect overall vegetable consumption in children [18,19,20].

Combining vegetables with another generally liked food (such as potatoes) could also influence vegetable consumption. Previous research has indicated that the size/form of vegetables [21], the variety of vegetables served [22], and the way that vegetables are served [23] can all have an effect on and increase vegetable consumption as part of school meals. Another method that has been explored is pairing vegetables with other foods. Correia et al. [24] served broccoli on a pizza, but ultimately found that this pairing did not increase vegetable consumption. Although it is clear that vegetable consumption can be increased by modifying methods of service, additional research needs to be conducted to explore whether or not pairing vegetables with other vegetables or meal components could increase vegetable consumption.

The school lunch venue may impact vegetable intake among children. School meals account for one third to a little over one half of the total calories consumed by children who participate in school lunches [25,26,27]. While current trends indicate that children are not consuming sufficient fruits and vegetables during their school meals [28,29], the USDA modifications to the National School Lunch Program (NSLP) implemented in 2012 to increase the nutritional value of meals (including more fruits and vegetables) [30,31] may be having a positive impact [32]. Furthermore, other school interventions have shown positive effects on behaviors and food choices in children, although interventions paired with other educational or environmental approaches tend to be more effective [33].

While multifaceted approaches are necessary to improve vegetable intake in children, exploring simpler strategies, based on previous research, may also be of value. In this study, we sought to examine how potatoes could act as a vehicle to increase the consumption of certain vegetables (mixed peas and carrots—MPACs). Potatoes were chosen as a relevant vehicle due to their general preference, recognition, and acceptability among children [34]. In 2013, Drewnowski and Rehm identified that French fries were the most commonly consumed vegetable in the NSLP [35]. Later research revealed that tater tots, along with French fries, were among the most frequently consumed vegetables in the NSLP [36]. Subsequently, after contacting multiple school districts in the Mountain Area (Utah, Idaho, and Colorado), NSLP providers indicated that a shaped potato face was the preferred potato product among most school-aged children. Given that tater tots contain 2–3 g of fat and 150 mg more sodium than fries and the shaped potato face, we opted to exclude them from this study. While French fries have a similar-to-slightly higher fat and sodium content compared to shaped potato faces, we selected the shaped potato product due to its greater popularity among survey participants. Recognizing that shaped potato faces can be considered a controversial food product due to their higher fat levels, we also developed a seasoned, diced potato product. This alternative had half the calories, one-fourth of the fat content of the shaped potato product, and lower sodium levels, providing an alternative potato option for exploration.

The choice of MPACs as the vegetables for this study was based on several factors. MPACs are vegetables commonly used in the NSLP, making it relevant for evaluating their consumption in school meals. Furthermore, MPACs were selected because they are easy to prepare and weigh, facilitating accurate measurements and evaluation during this study. Therefore, this study examined the extent that MPACs were consumed as a function of (1) the type of potatoes they were served with (shaped potato faces vs. diced) and (2) whether or not they were combined (same bowl vs. in a separate bowl). We hypothesized that combining MPACs with potatoes (both shaped potato faces or diced potatoes) would result in a higher MPAC consumption than the control condition or when served separately.

## 2. Materials and Methods

### 2.1. Study Design

All recruitment material, consent forms, questionnaires, and procedures were reviewed and approved by the Brigham Young University (BYU) Institutional Review Board. In addition, this study is available through the Open Science Framework. This study utilized a cross-over design to assess the impact of five different lunchtime meal conditions on MPAC consumption. Participants were provided with a base meal and one of five modified meal conditions (see below), with a minimum of one week between each condition. Each participant received the same meal on the same day, with the intervention meal order randomized over a span of six weeks (April–May 2022). The final week (week 6) was designated as a makeup day in which all meal interventions were served for participants that may have missed a particular session. MPAC consumption was assessed using plate waste (see below). Body composition, skin carotenoids, hand grip strength, fullness, and meal likeability were also assessed as additional variables in this study.

### 2.2. Participants

Children aged 7–13 (grades 1–6) were recruited through email invitations sent to parents and children registered in the Brigham Young University Sensory Laboratory database, as well as through word of mouth. To be eligible for participation, children had to be enrolled in school and participate in the NSLP at least twice a week. Additionally, they were required to have no food allergies, food restrictions, or health issues that would be impacted by the study meals to be served. Parents provided informed written consent for their child’s participation and assisted their child with answering questionnaires. Each child also provided assent to participate in the study. Both parents and children were informed of the purpose of the study and were financially compensated upon the completion of each meal condition.

### 2.3. Meal Conditions

Each meal included a base of 1% milk (Darigold, Seattle, WA, USA), chicken breast nuggets (Tyson Foods, Springdale, AZ, USA), unsweetened applesauce (TreeTop, Inc., Selah, WA, USA), and ketchup (House Recipe, Sysco Corp., Houston, TX, USA). As noted above, the vegetables used for this study were MPACs (Sysco Corp., Becker, MN, USA). The potato products used were shaped potato faces (McCain Foods USA, Inc., Oakbrook Terrace, IL, USA), or seasoned Sysco Imperial diced potatoes with the skins (Sysco Corp., Becker, MN, USA). The nutrient compositions for each food item served in this study are presented in Table 1.

**Figure 1:**
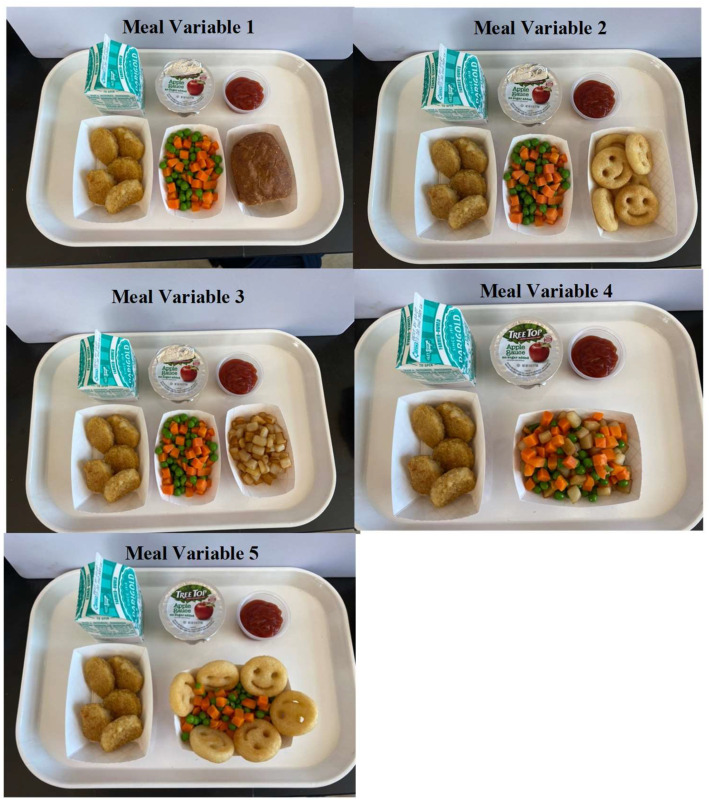
Meals conditions. Meal Variable 1—mixed peas and carrots (MPACs) and a whole-wheat bread roll served separately (control condition); Meal Variable 2—MPACs and shaped potato faces served in separate bowls; Meal Variable 3—MPACs and seasoned diced potatoes served in a separate bowl; Meal Variable 4—MPACs and seasoned diced potatoes served in the same bowl; Meal Variable 5—MPACs and shaped potato faces served in the same bowl.

**Figure 2:**
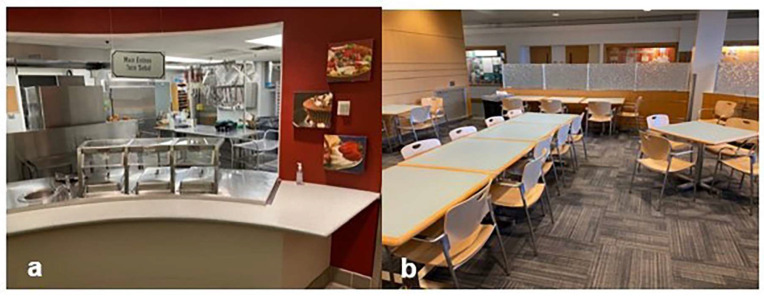
BYU’s Pendulum Court Cafeteria serving (**a**) and dining (**b**) areas, which assimilated a school cafeteria setting during this study.

The meal conditions included the base meal, plus (1) MPACs and a whole-wheat bread roll served separately (control condition), (2) MPACs and shaped potato faces served in separate bowls, (3) MPACs and seasoned diced potatoes served in a separate bowl, (4) MPACs and seasoned diced potatoes served in the same bowl, and (5) MPACs and shaped potato faces served in the same bowl (Figure 1). Each food component was weighed before and after each meal. The meals were provided in a food service lab designed to replicate a typical school cafeteria setting (Figure 2a,b), which also facilitated the evaluation of plate waste. The serving sizes were determined based on the serving recommendations of the NSLP (±10%; pre-weighed).

### 2.4. Procedures

During the initial visit, a member of the research team met with each participant and their parents to address any questions, ensure the completion of all consent and assent forms, and to initiate the study (check-in process). To maintain confidentiality, each participant was given a unique identifying number that was used throughout the study. After check-in, the participants and their parents proceeded to a room with a series of partitions (for privacy) for the measurement of body weight, height, and body composition. Following these measurements, participants proceeded to another partitioned area for the assessment of skin carotenoids and hand grip strength. The scores and results were not disclosed to the participants or parents to prevent any potential behavior changes that may influence study outcomes. These measurements were repeated during the participants’ final visit.

**Figure 3:**
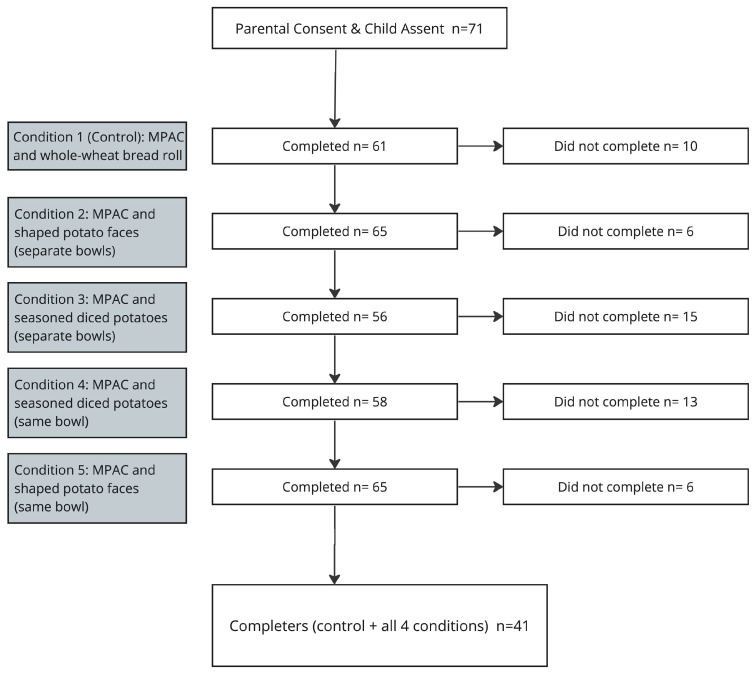
Flowchart illustrating the participation distribution across different meal treatments for the enrolled participants.

The meals were served in an on-campus cafeteria located on the BYU Provo, Utah, campus that included a serving line and free table seating for participants only to mimic a typical in-school cafeteria setting (Figure 2). Figure 3 outlines the participation numbers for each meal variable. Before receiving meals, parents/guardians were moved to a waiting area where they could observe their child without directly influencing their meal consumption. The participants were then asked to rate their current level of fullness (see below) and were provided an iPad/tablet with questions to gather information on their demographics, school lunch liking and weekly frequency, and the likeability of the lunch meal that day. Research assistants (RAs) were present during this phase to assist the participants with any questions and ensure the accuracy of the collected data. It was explicitly communicated to the research assistants that their role was to clarify questions for children and not to influence their responses or choices.

Participants were then given the pre-weighed meal, consisting of the variables described above, prepared in a commercial-grade food kitchen adjacent to the cafeteria. Each meal tray and the food and beverage items were weighed using a digital scale (Mettler-Toledo PR5001, Columbus, OH, USA). Each tray was assigned an identifier that was matched to the participant’s number for subsequent plate waste evaluation. During the meal, the participants were allowed to sit together if they wished but were instructed not to share food. At the conclusion of the meal, the participants again recorded their level of fullness, rated the overall likeability of their meal, and the amount of food given.

Under the guidance of an RA, each participant placed his/her finished meal tray on a cart for the subsequent determination of plate waste and returned the iPad/tablet to the RA. The participants were reunited with their parent/guardian, checked out by a study supervisor, and received a modest financial compensation.

Following the return of the meal trays, the food and beverage items were re-weighed using a digital scale to calculate the amount of each item consumed to the nearest 0.1 g. The potatoes were separated by hand from the MPACs when they were served together. The consumption of key nutrients was calculated using nutritional information provided by the food manufacturer of each product.

### 2.5. Measurements

#### 2.5.1. Demographics, Body Height, Weight, and Composition

At the participants’ first and last visit, a research assistant collected demographic information, including their age, sex, grade, and other evaluated endpoints, as listed below. This information was linked to the participants’ unique study ID.

The participants’ height, to the nearest 0.01 cm, was measured without shoes using a free-standing stadiometer (InBody BSM170, Seoul, Republic of Korea). Their body weight and composition (body fat percentage, fat mass in kg, and fat-free mass in kg) were assessed without shoes or socks using bioelectrical impedance (Tanita DC-430U, Arlington Heights, IL, USA). Their BMI (kg/m^2^) was calculated using their height and weight. The weight-for-age, height-for-age, and BMI-for-age z-scores were calculated based on the CDC growth charts for boys and girls 2 to 20 years [37]. Their body fat percentages were compared to the NHANES data from 1999 to 2004 to calculate z-scores based on their age and gender [38].

#### 2.5.2. Skin Carotenoids

Skin carotenoid levels, which serve as a biomarker of fruit and vegetable intake, were assessed using the VEGGIE METER^®^, a pressure-mediated reflectance spectroscopy (Longevity Link Corp, Salt Lake City, UT, USA). The scores obtained from this measurement range from 0 to 800, with higher values representing higher fruit and vegetable intake. Skin carotenoids have been identified as a valid non-invasive alternative to circulating blood carotenoids [39,40]. Measurements were conducted using standardized protocols developed by Whigham and Redelfs [41] and recently confirmed in recommendations by Radtke et al. [42], including cleaning the finger with a 70% alcohol swab, measuring the ring finger of the non-dominant hand to minimize staining, and calculating the mean of three scans.

#### 2.5.3. Hand Grip Strength 

The hand grip strength of the participants was measured using the Jamar^®^ Plus Hydraulic Hand Dynamometer (Eden Health, Bellingham, WA, USA). The measurements were conducted following the guidelines set by the American Society of Hand Therapists [43]. The participants were seated with both feet on the ground, and the arm of the hand being evaluated was positioned at a 90° angle. The participants squeezed the dynamometer until a constant reading was displayed. They then alternated to the other hand while maintaining proper form. This process was repeated three times on each side, and the average grip strength for each hand was recorded.

#### 2.5.4. Fullness and Likeability

**Figure 4:**
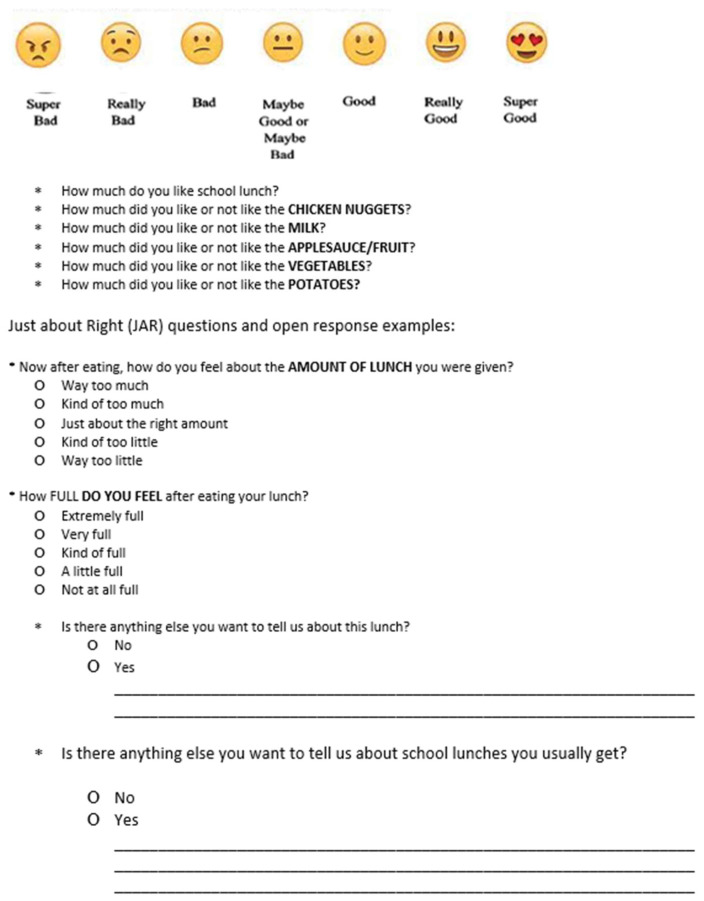
Seven-point facial and written hedonic scale with example questions.

At each visit, the participants assessed their pre- and post-meal fullness using the Freddy Fullness scale. This analog device, developed by Keller et al. [44] at Pennsylvania State University, was created as a way to help children understand and express their level of fullness. To assess likeability, participants were provided with an iPad or tablet linked to a list of questions using the Compusense20 software (Guelf, ON, Canada). The participants were asked to indicate what part of the school lunch was their favorite, and their feelings about the serving amount provided with the study before and after eating. The food liking questions were based on a finite 7-point facial and written hedonic scale: 1 = super bad, 2 = really bad, 3 = bad, 4 = maybe good or maybe bad, 5 = good, 6 = really good, and 7 = really good. Additionally, open-ended sections were included to allow the participants to provide additional comments if desired. A questionnaire example with the facial/written hedonic scale is provided in Figure 4.

#### 2.5.5. Plate Waste

Plate waste was evaluated to assess the preference and consumption of each meal component following the method described by Diktas et al. [19]. The remaining food (waste) on each plate was weighed to the nearest 0.1 g on a calibrated food scale and deducted from the initial pre-meal weight. Plate waste data play a critical role in this study as it provided insights into which food groups were eaten or discarded and the nutritional implications of these choices [45,46].

### 2.6. Power and Statistical Analysis

For statistical analysis, only data from participants that completed at least three of the five study conditions and completed both anthropometric evaluations were included in the final analysis. Repeated measures within a group ANOVA was used to calculate the statistical power for this study. Specifically, using a medium effect size (10 g difference, Cohen’s D effect size of 0.41), 0.5 correlation between tests, and *p* < 0.05, a total of 32 participants were needed to ensure 80% power.. Thus, our final sample size of *n* = 65 was sufficient. The original power analysis is available on the Open Science Framework (https://osf.io/mghd4/ accessed on 12 May 2022). This is a sample similar to other related studies [19]. A focus group was not conduced for this study as previouly described in the Open Science Framework.

Using the data, a linear mixed model with a random effect for participants was used to determine the differences in vegetable consumption based on the meal condition. The base model used to evaluate the differences in veggie consumption is defined as $y_{ij}=\mu +s_{i}+c_{j}+\epsilon_{ij}$, where $y_{ij}$ is the amount of veggies consumed by the *i*th participant under the *j*th condition, μ is the grand mean for consumption, $s_{i}$ is the random effect for the *i*th participant, $c_{j}$ is the effect of the *j*th condition, and $\epsilon_{ij}$ is an error term. This model used no covariates and simply compared conditions. Contrasts on the $c_{j}$ values were used to evaluate the impacts of a roll vs. potatoes (Contrast 1), combined service vs. separated service (Contrast 2), shaped potato faces vs. seasoned, diced potatoes (Contrast 3), and a service based on potato product interaction for the potatoes’ conditions (Contrast 4). To compare with the base model, we also consider a full model including covariates for age, sex, standardized height, standardized weight, standardized BMI, standardized body fat percentage, fullness, and standardized hand grip score. Model fitting was used to obtain a reduced model that optimized fit with parsimony using the Bayesian Information Criterion [47] and the Akaike Information Criterion [48].

## 3. Results

The participants’ characteristics are presented in Table 2. A total of 71 participants initially enrolled in this study. Four were removed for a failure to meet the participation criteria, and two for missing anthropometric data collected on the final visit, leaving 65 participants for the present analysis. Of the 65 participants, 66% (*n* = 43) completed all five conditions, 25% (*n* = 16) completed four conditions, and 9% (*n* = 6) completed three conditions. The average age of the participants was 9.75 ± 2.06 y with 57% boys and 43% girls.

Table 3 shows the MPAC consumption and overall nutrient consumption based on the meal condition. The mean vegetable consumption was 25.6 ± 27.6 g when shaped potato faces and MPACs were combined, 23.6 ± 28.2 g when diced potatoes and MPACs were served separately, 21.1 ± 30.4 g for the control condition, 20.8 ± 27.8 g when diced potatoes were combined with MPACs, and 16.8 ± 27.5 g when shaped potato faces were served separately. It should be noted that, despite instances where the standard deviation surpassed the corresponding mean, the data analysis revealed significant differences in MPAC consumption under these conditions (F = 5.20; *p* = 0.0005). The total kcal consumption ranged from 366 ± 103 kcal (seasoned diced potatoes served separately) to 452 ± 115 kcal (shaped potato faces combined with MPACs). The meal condition had a significant effect on kcal, protein, carbohydrate, fiber, total fat, saturated fat, and potassium consumption (*p* < 0.0001). Specifically, the consumption of these nutrients was generally higher when potatoes were a part of the meal compared to the roll condition. When the potatoes were served together in the same bowl (combined), there was a significant increase in the amount consumed, calories, protein, saturated fat, and potassium (*p* < 0.0001).

**Figure 5:**
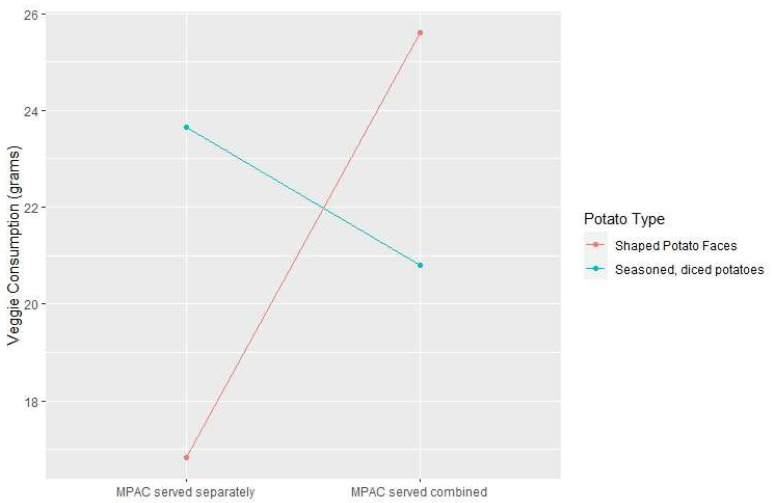
Interaction plot for the service (mixed peas and carrot vegetable consumption) by potato type interaction. LS means for the four conditions that include potatoes are plotted.

Table 4 shows the statistical contrasts for nutrients based on the meal condition variables. Meal condition was a significant predictor of MPAC consumption (F = 5.20; *p* = 0.0005), with MPAC consumption at its highest when combined with shaped potato faces in the same bowl (+8.77 g compared to serving MPACs and shaped potato faces in separate bowls) and lowest when combined with diced potatoes in the same bowl (−2.85 g compared to serving MPACs and diced potatoes in separate bowls). This interaction between potato type and combination status was highly significant (*p*-value < 0.0001); mixing potatoes with MPACs leads to increased MPAC consumption when the potato type is the shaped potato face, but leads to decreased MPAC consumption when the potato type is diced potatoes. This relationship is illustrated in Figure 5. The comparison between shaped potato faces and seasoned diced potatoes revealed significant differences in kcal, carbohydrate, fiber, total fat, saturated fat, sodium, and potassium consumption. The interaction between the potato type and combination status did not show significant effects on nutrient consumption, but the main effect for the combination status was significant for the total amount of food consumed (*p* = 0.0097), calories (*p* = 0.0491), protein (*p* = 0.0120), saturated fat (*p* = 0.0224), and potassium (*p* = 0.0128). This suggests that the way the food was served had a significant impact on several nutrients, except for sodium levels.

Table 5 lists the predictors used in the best-fitting statistical model for MPAC consumption. The full (original) model also included terms for weight for age (z-score), BMI for age (z-score), food likeability, fullness, and hand grip strength (z-score), but none of these provided a significant explanatory ability about MPAC consumption above and beyond that provided by the model described in Table 5. The model with the best fit in terms of BIC and AIC included terms for age, sex, standardized height, and standardized body fat. A maximum-likelihood-based test indicated that our best-fitting model had a significant explanatory ability (*p* < 0.0001). Older children consumed more vegetables than their younger counterparts (*p* = 0.0007) and males consumed more vegetables than females (*p =* 0.0099). There was a positive relationship between standardized height and vegetable consumption (*p* = 0.0065), and a negative relationship between standardized body fat and vegetable consumption (*p* = 0.0153). These findings highlight the importance of age, sex, body fat, height, and the specific condition in influencing the amount of vegetables consumed by the participants.

The significance of carotenoids was observed only after eliminating factors such as body fat and the interaction between body fat and age (*p* = 0.0012). However, this relationship was very similar to the association between body fat and vegetable consumption (*p* = 0.03), indicating that a higher body fat percentage was associated with lower vegetable consumption. It is important to note that, due to instrument/calibration errors, the carotenoid data from the last visit could not be confidently used and may have influenced the correlation and final data analysis.

## 4. Discussion

This study demonstrated that incorporating potato products can be an effective strategy for increasing overall vegetable consumption in a typical school meal, with the most significant consideration being the important interaction between combination status and potato products; combining MPACs with potatoes is helpful to MPAC consumption when using shaped potato faces but has a small negative impact on MPAC consumption when using diced potatoes. These results were surprising, as during informal focus groups, after individuals tried the seasoned, diced potatoes, they were generally preferred over the shaped faces. The lack of familiarity of the seasoned, diced potatoes in the main study, and the general hesitancy of children to try new foods, could have contributed to their negative impact on MPAC consumption. While shaped potato faces are commonly found on school menus, there may be concerns about them contributing to increases in calorie and fat intake. When comparing the bread roll to the shaped potato faces, potato faces contained approximately 50 more calories and 6 g of fat. However, most of these fats were unsaturated fats, which are considered a healthier fat option.

It is worth noting that potatoes have sometimes been associated with being “unhealthy” due to certain preparation methods involving deep-frying or an excessive use of butter or cheese [49]. However, there are alternative approaches to address these concerns. In this study, our seasoned, diced potatoes reduced the amount of added fat, making it equivalent to the control (wheat roll) at 1.5 g of fat per serving, while reducing sodium by 32 mg. When the seasoned, diced potatoes were served separately from the MPACs, an increase in overall vegetable consumption was observed, aligning with patterns seen in high-school-aged students [50]. As previously mentioned, while seasoning and taste did not seem to significantly impact vegetable consumption in young children, it seems to make a difference in high-school settings [19,50]. This suggests that taste preferences and palatability may change as children grow older.

Furthermore, a study conducted in US elementary schools demonstrated that children increased their fruit and vegetable consumption when an additional fruit or vegetable was added to the menu. This finding aligns with our own study, where combining potatoes with the MPACs introduced an additional vegetable to the plate/meal and resulted in increased vegetable consumption. Given the success of shaped potato faces in promoting vegetable intake, future research could explore the potential combination of shaped potato products with other vegetables to further enhance consumption. Another approach worth considering is consistently offering a variety of vegetables to preschool children, as demonstrated by Ahern et al. [18], who found that this approach led to an overall increase in vegetable consumption. While concerns about energy intake versus nutrient content persist, studies have shown that potatoes, in general, do not induce children to seek out more energy-dense foods. In fact, Akilen et al. [51] found that boiled mashed potatoes and French fries helped promote appetite control and satiety in children, as indicated by ghrelin and peptide YY levels. Although the potato mediums used in our study were different, these additional dynamics warrant further investigation.

Referring to the nutrient breakdown charts presented earlier, the differences in the calories and fat content between the intervention meals were minor. Potatoes provide key nutrients such as potassium, magnesium, iron, fiber, carbohydrates, vitamin C, and other phytochemicals, which are not as abundant in a conventional wheat roll [49]. This study focused on analyzing the differences in macronutrients among the meals offered, and further research is needed to compare the actual caloric intake of the foods consumed in this study versus the foods offered in a school setting. Additionally, investigating the cost–benefit relationship of substituting potatoes for a conventional wheat roll would be valuable.

The difference in vegetable consumption between seasoned, diced potatoes with MPACs served separately and shaped potato faces served combined with MPACs, approximately 9 g, may be considered relatively small in a practical context. Nonetheless, even small steps towards improving vegetable consumption are significant for enhancing nutrition intake. Furthermore, the underlying reasons for the differential impact of combination status between seasoned diced potatoes and shaped potato faces remain unknown. It is plausible to suggest that the preference for shaped potato faces, which are more familiar and commonly served in school cafeteria lunches, contributed to their higher acceptance compared to the less-familiar seasoned diced potatoes. Exploring this aspect, particularly considering the reluctance of younger children to try new foods, could be an interesting avenue for future research [52,53].

It is important to recognize that limiting variables that potentially impact meal consumption in school settings were not explored within this study. Factors such as the duration of mealtimes, the alignment of lunch with recess, and the integration of nutrition education and interactive activities could significantly influence overall meal consumption and fruit and vegetable intake [54,55,56,57]. Additionally, this study exclusively examined two specific potato types, potentially disregarding effects that could arise with variations like French fries or mashed potatoes. Given the focus on children, their behavior emerged as a potential study limitation. It is worth noting that, despite participants being advised to abstain from eating for a minimum of two hours before the session, the Freddy Fullness survey revealed instances where this timeframe was not adhered to, and although instructed to sample each component, certain participants exhibited non-compliance due to existing biases against vegetables.

However, employing a cross-over design enhanced the internal validity by exposing each participant to all experimental conditions, reducing confounders and enhancing the result reliability. Additionally, the study’s favorable sample size enabled robust statistical analyses and the examination of various measures. Furthermore, rigorous control over anthropometric assessments and meal tracking, a challenge in studies with children, fortified the study’s methodology. Lastly, objective measures like plate waste assessment bolstered accuracy by minimizing subjective reporting reliance.

## 5. Conclusions

In conclusion, this study has provided valuable insights into the potential benefits of incorporating potatoes in school meals to increase additional vegetable consumption. The findings indicate that the inclusion of shaped potato faces, a product commonly served in school meals, significantly increased additional vegetable consumption among children, but only when they are mixed with vegetables; serving the vegetables separately from the shaped potato product was associated with a reduced consumption of vegetables. This demonstrates the effectiveness of leveraging the appeal of certain food items to encourage healthier eating habits. While we are not promoting shaped potato faces as a nutritious component of a healthy diet, this study indicates that there needs to be further research on leveraging other foods to increase vegetable consumption. Additionally, this study highlights the importance of considering the potential interaction between the type of potato product and the combination status (served in the same bowl or separate bowl) in influencing vegetable consumption. The results suggest that combining shaped potato faces with vegetables in the same bowl increases vegetable intake. However, as indicated, further research is needed to explore these differences in service and product interactions.

Additionally, potatoes are not only a popular staple food, but are also classified as a vegetable due to their nutrient content and botanical classification. By incorporating potatoes as part of the meal options, the children had the opportunity to increase their overall vegetable intake and receive the associated nutritional benefits. This finding underscores the importance of recognizing potatoes as a vegetable and highlights their potential contribution to promoting healthier eating habits in children. However, caution should be exercised to avoid introducing excessive amounts of fat and sodium through certain potato preparations and options.

Overall, this study emphasizes the potential of potatoes as a means to increase vegetable consumption in school meals and provides valuable insights for developing strategies to promote healthier eating habits among children.

## Figures and Tables

**Table 1:** Amounts and nutrient composition of each food component served at meals.

Meal Nutrients							
	Peas/Carrots Mix (Seasoned) ^a^	Whole Wheat Roll	Shaped Potato Face	Seasoned Diced Potatoes ^b^	1% Milk	Chicken Nuggets	Apple Sauce
Amount (g)	85	40	85	85	260	100	110
Calories	54	110	160	75	110	250	60
Protein (g)	2.9	4	2	2	8	13	1
Carbohydrates (g)	9.5	17	22	15	13	14	15
Fiber (g)	2.9	3	2	1.2	0	0.8	3
Total Fat (g)	1.1	1.5	8	1.5	2.5	16	0
Sat Fat (g)	0.2	0	1	0.2	1.5	3	0
Sodium (mg)	147	190	170	158	130	630	0
Potassium (mcg)	165	109	310	360	400	160	116

^a^ Each batch of MPACs (2 kg) was seasoned with 2 g of salt, 2 g of pepper, and 20 g of margarine. ^b^ Each batch of diced potatoes (2.7 kg) was seasoned with 13 g of kosher salt, 3 g of rosemary, 3 g of black pepper, 0.5 g of garlic powder, 1 g of onion powder, and 45 mL of olive oil. These seasonings were added to improve palatability. No additional salt was added to the shaped potato face.

**Table 2:** Participant characteristics and t-test results by sex.

	Total (*n* = 65)	Girls (*n* = 28)	Boys (*n* = 37)	T-Statistic (Girls vs. Boys)	*p*-Value
Age	9.8 ± 2.06	9.5 ± 2.05	9.9 ± 2.08	−0.86	0.3921
Weight (kg)	37.1 ± 11.9	34.1 ± 10.9	39.3 ± 14.6	−1.60	0.1157
Height (m)	1.43 ± 0.16	1.41 ± 0.15	1.45 ± 0.16	−1.06	0.2925
Weight z-score	0.42 ± 1.00	0.18 ± 0.86	0.60 ± 1.08	−1.68	0.0987
Body fat (%)	18.4 ± 5.79	19.9 ± 5.51	17.3 ± 5.82	1.79	0.0779
BMI (kg/m^2^)	17.5 ± 2.93	16.8 ± 2.31	18.1 ± 3.24	−1.80	0.0769
Hand grip Z-score	−0.58 ± 0.89	−0.61 ± 0.88	−0.55 ± 0.92	−0.27	0.7890
BMI z-score	0.08 ± 1.04	−0.15 ± 0.95	0.25 ± 1.08	−1.57	0.1212
Grip strength (kg)	14.11 ± 5.21	13.18 ± 5.42	14.81 ± 5.01	−1.25	0.2162
Fullness (pre-meal)	5.02 ± 2.64	4.83 ± 2.69	5.17 ± 2.63	−0.51	0.6120
Fullness (post-meal)	11.27 ± 1.04	11.85 ± 2.88	10.84 ± 2.26	1.59	0.1176
Fullness (difference)	0.08 ± 1.04	7.02 ± 3.05	5.67 ± 2.65	1.91	0.0613

Mean ± standard deviation.

**Table 3:** LS means and standard deviations for vegetable and nutrient consumption by meal conditions.

Nutrient Consumption	Control (Roll)	Shaped Potato Faces Served Separately	Diced Potatoes Served Separately	Dice Potatoes Served Combined	Shaped Potato Faces Served Combined	F	*p*
MPACs (g)	21.1 ± 30.4	16.8 ± 27.5	23.6 ± 28.2	20.8 ± 27.8	25.6 ± 27.6	5.20	0.0005
MPACs (kcal)	13.4 ± 19.3	10.7 ± 17.5	15.0 ± 17.9	13.2 ± 17.7	16.3 ± 17.5		
Nutrient Consumption Amount (g)	355 ± 151	354 ±153	345 ± 159	361 ± 155	377 ± 148	2.52	0.0418
Calories (kcal)	414 ± 107	432 ± 122	366 ± 103	374 ± 102	452 ± 115	28.25	<0.0001
Protein (g)	20.2 ± 5.6	17.7 ± 5.6	17.9 ± 5.4	18.4 ± 5.3	18.8 ± 5.2	10.10	<0.0001
Carbohydrate (g)	44.0 ± 14.9	45.7 ± 15.5	38.5 ± 14.9	39.4 ± 14.7	47.9 ± 15.2	20.51	<0.0001
Fiber (g)	5.7 ± 2.4	4.7 ± 2.1	4.2 ± 1.9	4.1 ± 1.9	5.0 ± 2.1	23.76	<0.0001
Total Fat (g)	17.1 ± 3.2	21.0 ± 5.2	16.6 ± 3.7	16.8 ± 3.5	21.8 ± 4.8	60.77	<0.0001
Saturated Fat (g)	3.6 ± 0.8	4.0 ± 1.1	3.5 ± 0.9	3.6 ± 0.9	4.2 ± 1.1	24.87	<0.0001
Sodium (mg)	791 ± 175	748 ± 192	727 ± 185	739 ± 173	785 ± 173	5.07	0.0006
Potassium (mcg)	477 ± 206	651 ± 265	616 ± 312	646 ± 294	695 ± 254	31.38	<0.0001

Note 1: LS means estimate the averages that would have been seen if the data had been balanced (i.e., the same mix of covariates in each group). Note 2: LS means reported for MPACs are obtained from the statistical model illustrated in Table 4. All other means are LS means from the mixed effects linear model with the condition as the only fixed effect. SD values are raw standard deviations (not estimated standard errors).

**Table 4:** *p*-values for the condition effect and estimated LS mean differences and *p*-values for statistical contrasts of interest when comparing MPAC consumption and overall nutrient consumption across meal conditions.

	Overall Condition Effect	Potatoes Minus Roll	Comb. Minus Separate	Shaped Potato Faces Minus Diced	Potato Type by Combination Status Interaction Effect
MPACs (g) *p*-value	(*p* = 0.0005)	0.60 g (*p* = 0.7100)	2.96 g (*p* = 0.0422)	−1.01 g (*p* = 0.4879)	11.62 g (*p* < 0.0001)
MPACs (kcal) *p*-value	(*p* = 0.0005)	0.38 kcal (*p* = 0.7100)	1.88 kcal (*p* = 0.0422)	−0.64 kcal (*p* = 0.4879)	7.38 kcal (*p* < 0.0001)
Nutrient Consumption Amount (g)	(*p* = 0.0418)	4.38 g (*p* = 0.6066)	19.83 g (*p* = 0.0097)	12.67 g (*p* = 0.0972)	7.48 g (*p* = 0.6233)
Calories (kcal)	(*p* < 0.0001)	−8.11 kcal (0.2988)	13.76 kcal (*p* = 0.0491)	72.13 kcal (*p* < 0.0001)	11.60 kcal (*p* = 0.4057)
Protein (g)	(*p* < 0.0001)	−2.01 g (*p* < 0.0001)	0.79 g (*p* = 0.0120)	0.10 g (*p* = 0.7401)	0.62 g (*p* = 0.3201)
Carbohydrates (g)	(*p* < 0.0001)	−1.14 g (*p* = 0.2547)	1.56 g (*p* = 0.0819)	7.86 g (*p* < 0.0001)	1.19 g (*p* = 0.5050)
Fiber (g)	(*p* < 0.0001)	−1.12 g (*p* < 0.0001)	0.12 g (*p* = 0.3352)	0.73 g (*p* < 0.0001)	0.36 g (*p* = 0.1610)
Total Fat (g)	(*p* < 0.0001)	1.91 g (*p* < 0.0001)	0.51 g (*p* = 0.1171)	4.75 g (*p* < 0.0001)	0.61 g (*p* = 0.3541)
Saturated Fat (g)	(*p* < 0.0001)	0.30 g (*p* < 0.0001)	0.15 g (*p* = 0.0224)	0.56 g (*p* < 0.0001)	0.09 g (*p* = 0.4651)
Sodium (mg)	(*p* = 0.0006)	−41.72 mg (*p* = 0.0035)	23.80 mg (*p* = 0.0611)	33.65 mg (*p* = 0.0083)	24.79 mg (*p* = 0.3280)
Potassium (mcg)	(*p* < 0.0001)	175.15 mcg (*p* < 0.0001)	32.28 mcg (*p* = 0.0128)	42.47 mcg (*p* = 0.0047)	14.15 mcg (*p* = 0.6349)

Note 1: Column one gives the *p*-value for the F test comparing the five conditions. In columns 2 through 5, we report the estimated LS mean effect size associated with each statistical contrast (comparison) and the associated *p*-value. Note 2: MPACs = mixed peas and carrots. Note 3: The potato type based on the combination status interaction effect in the fifth column is the effect for combining potatoes with MPACs when the potato type is the shaped potato face minus the effect for combining potatoes with MPACs when the potato type is diced potatoes. For example, when the outcome is MPACs in grams (top row) and using shaped potato faces, changing from potatoes served separately to potatoes and MPACs combined, the increase in MPAC consumption is 8.77 g. When using diced potatoes, the comparable effect for combining potatoes with MPACs is −2.85 g. The difference between these two effects is 11.62 g. Note 4: LS means reported for MPACs are obtained from the statistical model illustrated in Table 4. All other means are LS means from the mixed effects linear model with the condition as the only fixed effect. SD values are raw standard deviations (not estimated standard errors).

**Table 5:** Model predictors of vegetable consumption.

Predictor	Correlation w/Vegetable Consumption	Coefficient	F	*p*	Likelihood Ratio Statistic	*p*
Overall Model Significant factors:					49.1	<0.0001
Age	0.38	4.43	12.74	0.0007		
Sex	na	13.86	7.06	0.0099		
Height (z-score)	0.30	5.63	7.92	0.0065		
Body Fat (z-score)	−0.15	−11.44	6.20	0.0153		
Condition	na	na	5.20	0.0005		

## Data Availability

Any data not found at in the Open Science Framework (https://osf.io/mghd4/ accessed on 12 May 2022) will be made available upon request.
